# Unproven stem cell–based interventions and achieving a compromise policy among the multiple stakeholders

**DOI:** 10.1186/s12910-015-0069-x

**Published:** 2015-11-04

**Authors:** Kirstin R. W. Matthews, Ana S. Iltis

**Affiliations:** Center for Health and Biosciences at the Baker Institute for Public Policy, 6100 Main Street MS40, Houston, TX 77005 USA; Department of Philosophy and Center for Bioethics, Health and Society, Wake Forest University, 1834 Wake Forest Road, Winston-Salem, NC 27106 USA

**Keywords:** Stem cells, Clinical trials, Patient advocacy, Expanded access, Stem cell-based interventions, Stem cell tourism

## Abstract

**Background:**

In 2004, patient advocate groups were major players in helping pass and implement significant public policy and funding initiatives in stem cells and regenerative medicine. In the following years, advocates were also actively engaged in Washington DC, encouraging policy makers to broaden embryonic stem cell research funding, which was ultimately passed after President Barack Obama came into office. Many advocates did this because they were told stem cell research would lead to cures. After waiting more than 10 years, many of these same patients are now approaching clinics around the world offering experimental stem cell-based interventions instead of waiting for scientists in the US to complete clinical trials. How did the same groups who were once (and often still are) the strongest supporters of stem cell research become stem cell tourists? And how can scientists, clinicians, and regulators work to bring stem cell patients back home to the US and into the clinical trial process?

**Discussion:**

In this paper, we argue that the continued marketing and use of experimental stem cell-based interventions is problematic and unsustainable. Central problems include the lack of patient protection, US liability standards, regulation of clinical sites, and clinician licensing. These interventions have insufficient evidence of safety and efficacy; patients may be wasting money and time, and they may be forgoing other opportunities for an intervention that has not been shown to be safe and effective. Current practices do not contribute to scientific progress because the data from the procedures are unsuitable for follow-up research to measure outcomes. In addition, there is no assurance for patients that they are receiving the interventions promised or of what dosage they are receiving. Furthermore, there is inconsistent or non-existent follow-up care. Public policy should be developed to correct the current situation.

**Conclusion:**

The current landscape of stem cell tourism should prompt a re-evaluation of current approaches to study cell-based interventions with respect to the design, initiation, and conduct of US clinical trials. Stakeholders, including scientists, clinicians, regulators and patient advocates, need to work together to find a compromise to keep patients in the US and within the clinical trial process. Using HIV/AIDS and breast cancer advocate cases as examples, we identify key priorities and goals for this policy effort.

## Background

Significant public debate about stem cell (SC) research started in 1998 after the first human embryonic SC line was first successfully grown in a lab [1]. Heated debates occurred during the presidential elections in 2000 and 2004 about expanding federal funding for embryonic SC research and increasing the number of embryonic SC lines eligible for this funding. Scientists strongly advocating for more funding and less regulation were joined by a large number of patient advocate groups, such as the Juvenile Diabetes Research Foundation (JDRF) and the Michael J. Fox Foundation for Parkinson’s disease. Numerous grassroots organizations joined the efforts as well as business leaders such as Robert Klein, whose efforts in California helped pass Proposition 71 that created the California Institute of Regenerative Medicine (CIRM). Scientists and patient advocates promoted the research and therapeutic potential of all SC types to treat debilitating diseases. Media coverage regularly extolled the promises of SC research [2].

Unfortunately, the growing publicity on the field had negative consequences—unproven SC-based intervention (SCBI) clinics flourished. Starting in the mid-2000s, clinics began exploiting the popularity of SCs to market and selling SCBIs which have not undergone clinical trials. Clinics emerged claiming to cure diseases ranging from autism to multiple sclerosis [3–5]. While some patients find clinics within the US offering unproven SCBIs, many travel abroad, often to countries with less developed medical infrastructures and regulatory oversight, a concept or practice often referred to as SC tourism.

Patients frustrated by what they saw as the slow progress of science and desperate for a cure began paying for access to unproven interventions despite strong discouragement from scientists and doctors [3, 6–9]. Although clinical trials utilizing SCs have increased, the majority are still in the safety-testing stage involving a limited number of patients [10]. And many patients have conditions that are not part of a current SC trial. Other patients want more than research; they hope for a cure or at least an opportunity for some improvement now [9]. Clinic advertisements successfully use a “rhetoric of hope” to appeal to patients, and patients respond positively when probed by researchers saying the experience increases their happiness and hope in the future [9]. Even more shocking is that many SC tourism patients are minors, under the age of 18 (more than 40 % by some calculations) who had limited ability to participate in the decision-making process [7, 8]. Although we cannot know truly what is being offered by which clinics and the number of patients who travel to obtain SCBIs, there is evidence that SC tourism is a substantial and increasing industry [5, 11–14]. And it is still increasing, despite efforts to combat it [15].

As early as 2008, scientists and policy scholars became concerned about the negative impact these clinics would have on patients as well as the field [16–19]. For patients seeking SCBIs, the costs and risks associated with SC tourism are significant [3]. The average cost is $47,000 per treatment, but clinics charge from $3500–$400,000, and often require travel, which adds to the expense [20]. If patients are harmed from the procedure, it is unclear in the US whether or not third party payers will cover the cost of treating side-effects [21].

Patients seeking unproven SCBIs may not be adequately informed about the risks associated with these interventions and the very limited prospect of benefit [22–24]. In fact, opinion polls indicate the public believes the benefits outweigh perceived risks [25]. Patients’ hope for improvement may be a powerful factor in decision making and they may not be dissuaded by information about the lack of safety and efficacy data [8, 26, 27]. Clinics in the US most commonly offer treatments related to pain management or improved healing of an existing injury although there is no strong scientific evidence that these treatments are beneficial [28]. Clinics overseas offer more extensive interventions, culturing autologous SCs or even injecting human embryonic SCs. As a result, several patients have developed serious conditions or died from unregulated procedures. An Israeli boy with a rare genetic brain disease developed tumors after obtaining fetal SC injections in Russia and a girl treated in Costa Rica for multiple sclerosis experienced catastrophic demyelinating encephalomyelitis following a stem cell transplantation [29, 30]. A woman with lupus nephritis died in 2010 after receiving a SC procedure in Bangkok [31]. And a clinic in Germany, XCell Center, was shut down after the death of a Romanian baby after SC injections into the brain [32].

It is often unclear, even to the patients, what cells are being provided, if the procedure has been standardized and what effects it has in both the short- and long-term. This can render patients ineligible to receive other treatments or participate in future clinical research [23]. The lack of follow-up post-treatment also makes it impossible to identify long-term risks. Providing unproven SCBIs without adhering to a protocol and systematically collecting data leaves scientists and the public with little to no useful new knowledge about the safety and efficacy of these interventions and undermines the interests of future patients [22]. Even when clinics claim to be conducting research, the rigor and reliability of their data are questionable [24]. Most clinics offer testimonials by alleged patients and clinic representatives as data, which do not provide reliable evidence necessary to advance the field [5]. SC tourism undermines the interest of future patients causing research to be less rigorous; when widespread early access to treatments is provided patients are less likely to participate in trials [23, 33].

It also is difficult for patients to receive proper medical attention after a SCBI since the nature of their original treatment might be unknown. Furthermore, most clinics are located in countries where standards for patient care are below what US patients expect and where patients lack protections afforded by liability standards and other requirements that are standard in the US [5].

One of the major science organizations, the International Society for Stem Cell Research (ISSCR), developed guidelines to distance the scientific community and SC research from clinics offering unproven and unregulated SCBIs. Developed in 2008 with an updated draft circulating for comment in 2015, these guidelines support the traditional clinical trial regulatory and ethical oversight process and make recommendations on how scientists should translate research into the clinic regardless of the country where their research is being conducted [16, 34]. Researchers, bioethicists, physicians, funders and regulators are concerned that the safety and efficacy of SCBIs remain unknown. They also note that the procedures are not well-documented and there is a lack of transparency regarding what patients receive. Furthermore, scientists worry that fraudulent clinics will negatively impact the reputation of the field, much like they worry that scientific misconduct will harm public perception of science [35-37]. If patients are harmed, regulators and the public might link negative consequences to SC and regenerative medicine research, threatening funding and perhaps leading to further restrictions on such research.

In the US, the Food and Drug Administration (FDA) regulates clinical trials and approves drugs, biologics, and medical devices, among other things [37]. The FDA’s role in the emergence of SC tourism is complex [33]. In the early days of debates regarding SCBIs, it was unclear whether the FDA had regulatory authority over autologous SCBIs, which typically involve using the patient’s own cells from one site, such as bone marrow or adipose tissue, and injecting them into the location of the diseased or injured tissue [28]. These procedures make up the majority of SCBIs offered in US clinics. The FDA’s authority was debated because the donor and recipient were the same person [38]. Some advocates argued that a patient’s own cells should not be considered drugs or biologics subject to FDA oversight; their use should be “treated as a medical practice” and left to the discretion of physicians [22]. They described the idea of FDA regulating autologous SC interventions as “your cells = drugs” and decried it as illogical as well as fatal for innovation.

The FDA asserted its authority and investigated clinics that offered SCBIs which utilized more than “minimally manipulated” cells [38]. This included autologous procedures, especially if the cells were processed and expanded in culture. The disputes between the FDA and clinics offering these services led to the defining case, *United States v Regenerative Sciences LLC* (Regenexx) [39]. The DC Circuit Court of Appeals ruled that the “Regenexx procedure,” an intervention which involved culturing and expanding a patient’s autologous SCs before reimplantation, was subject to FDA regulation (Regenexx.com). The court found that the procedure involved more than “minimal manipulation” of the cells and the company “violated federal laws regulating the manufacture and labeling of drugs and biological products by producing, as part of their medical practice, a substance consisting of a mixture of a patient’s SCs and the antibiotic doxycycline” [39].

Many scientists considered this ruling an important step toward ensuring that only safe and effective SCBIs are offered to patients [40]. The court decision led to the shut-down of many US SCBI clinics, although some still are open [28]. Unfortunately, rather than prompting clinical research and widespread efforts to demonstrate safety and efficacy of SCBIs through clinical trials, the ruling drove, more than ever, providers and patients to SC tourism. The FDA only regulates products and their uses in the US, so several clinics relocated to countries without strict oversight of cell-based therapies. For example, Regenexx provides their cultured SC procedure in the Cayman Islands and another clinic, Precision Stem Cell, recruits in the US for a Colombian clinic [28, 40]. SC tourism is especially prevalent in Latin America, Asia and the Caribbean [22].

The current landscape in which patients travel to unregulated clinics outside of the US to obtain unproven SCBIs poses a number of serious concerns for individual patients as well as for society, particularly for future patients. In this paper, we argue that public policy should be developed to reduce SC tourism. Policy should be aimed at bringing patients home and fostering responsible scientific research as well as access for patients. This will require discussions about alternative approaches to the design and conduct of clinical trials as well as to how interventions are approved by FDA.

SCs and regenerative medicine were not the first areas of medical research to be seen pitting advocates, who wanted widespread access to experimental interventions, against scientists and regulators, who wanted to evaluate the safety and efficacy of these interventions before making them widely available. Two examples with different policy responses and outcomes were HIV/AIDS activists in the 1980s and breast cancer advocates in the 1990s. Using these cases as examples, we identify key priorities and goals for the SC tourism policy effort and essential steps in the policy development process.

## Discussion

### HIV/AIDS and breast cancer advocacy

The struggle between patients and regulators is not unique to SC research. HIV/AIDS activism in the 1980s and breast cancer activism in the 1990s reflect disconnects that can occur in medicine between patients’ desires for access to new interventions and scientists, regulators or third party payers trying to make sure interventions are safe and effective prior to widespread marketing and use. In these two examples, advocates sought to obtain access to therapies prior to the completion of clinical trials, while regulators and third party payers asked for patience. Each secured early access but it did not necessarily result in the best outcomes for the patients. Important differences between them should inform the policy approach to address SC tourism.

In the 1980s, HIV/AIDS was a devastating disease, killing millions worldwide [41]. Desperate for a treatment, AIDS activists argued that the FDA approval process was too long and inappropriate for fatal conditions such as AIDS. At the time, the FDA had informal programs that allowed some patients access to investigational new drugs (INDs). AIDS activists pushed the FDA to formalize these programs. First, the 1987 changes to the IND program established mechanisms by which patients could obtain INDs outside of clinical trials [42]. Then, the 1988 fast-track initiative allowed for expedited approval of some drugs for conditions such as AIDS. In 1990, the parallel track program allowed HIV/AIDS patients who could not enroll in clinical trials access to experimental drugs [43]. In addition, in 1992 the FDA allowed the use of surrogate endpoints as a basis for drug approval to accelerate the process [33]. Surrogate endpoints are alternative measures which correlate with the outcome that is of primary interest but can be measured more readily. An example of a surrogate endpoint is tumor shrinkage rather than longer survival. How confident we must be that a surrogate endpoint is a valid substitute for the endpoint of primary interest is debated [44, 45].

These policy changes were prompted by direct activism, including protests in front of FDA headquarters [33]. Activists were aware that drugs were being approved abroad, especially in Japan, and found ways to import and distribute them to AIDS patients in the US [46]. Patients enrolled in clinical trials would use other experimental agents acquired outside of studies while in trials and they sometimes shared study pills, compromising data integrity [47]. These factors helped encourage changes within the FDA to promote research while facilitating access.

A second example of a disconnect between scientists, patients and regulators revolved around access to and insurance coverage for high dose chemotherapy (HDC) and autologous bone marrow transplantation (ABMT) to treat metastatic breast cancer in the 1990s. HDC/ABMT had not been proven to be safe and effective for breast cancer, but it had been used successfully in other types of cancers and preliminary studies suggested it might be effective in breast cancer. Patients and oncologists were eager to try this intervention despite its high cost and lack of evidence [48, 49].

Unlike the early HIV/AIDS medications, the drugs here were approved, albeit for other uses. The advocates were not fighting the FDA for access [50]. Instead they were trying to get third party payers to cover the treatment. Initially, many third party payers, both private insurance companies and government programs, refused coverage because it was experimental. To gain access, patients and families headed to court, suing insurance companies to demand coverage or to recover damages when patients died after having been denied coverage for HDC/ABMT [43]. Results varied, but many third party payers began covering HDC/ABMT for women with advanced breast cancer as a result of the litigation [50, 52, 53].

In part as a result of the successful demands for coverage outside of clinical trials and the perception among some patients and oncologists that HDC/ABMT was an appropriate treatment for metastatic breast cancer, it took an inordinate amount of time to complete the randomized controlled trials that evaluated the safety and efficacy of the intervention. Patients wanted to receive treatment, not risk being in the control group of a trial. Study results eventually showed that HDC/ABMT did not improve survival and in some cases led to decreased life-span and increased discomfort compared to standard treatment [54, 55]. Not only did patients pursue an ineffective intervention at great expense to third party payers, but in some cases the intervention caused them more harm.

### Lessons from HIV/AIDS and breast cancer

Advocate movements to facilitate access to experimental interventions for HIV/AIDS and breast cancer contain lessons that should inform patients, SC advocates, scientists, and regulators thinking about SC tourism. While the HIV/AIDS and breast cancer case studies focus on one disease, SCs are being utilized to treat a broad range of medical conditions. Moreover, unlike the HDC/ABMT (but like the HIV/AIDS) case, SC tourism involves many different interventions. Despite any differences, learning from previous activist movements and policy responses can help guide us in thinking about how to address SC tourism.

The AIDS activists collaborated effectively with clinicians, scientists and regulators to improve policies at the FDA. They found ways to improve the system but still worked with the system. The FDA listened and acknowledged the values, priorities and goals of the activist groups. The resulting compromises in FDA regulations allowed HIV/AIDS scientists and clinical researchers to continue to evaluate potential medications for safety and efficacy, while patients obtained early access. Measures were in place to collect data over the long-term to meet the societal interest in ensuring the safety and efficacy of drugs marketed and sold in the US. Rather than ignoring advocates’ pleas, the FDA collaborated with multiple stakeholders to develop policies that would address the interests of different stakeholders. As a result, they compromised and developed a series of policies to allow quicker access to patients while still honoring the research process. In addition to conducting studies that did not use placebo controls, other compromises included the use of surrogate endpoints, such as reduced CD4 counts, and early termination of studies based on preliminary data [56, 57].

One negative result of these compromises, which should be acknowledged in developing SC policy, was that after access to experimental drugs was granted, many of the clinical studies were less rigorous [58]. Because of the deadly nature of the disease, patients did not want to risk receiving a placebo or standard-of-care, which were not very effective. Therefore enrollment in trials decreased taking longer to finish the studies. This reflected a balancing of interests and priorities. But these outcomes were an improvement over the previous situation where untested drugs were being used by patients and experimental pills were being shared, compromising clinical trial data integrity.

Ultimately, a collaborative approach was essential in HIV/AIDS to advance the different goals of patient advocates, scientists, clinicians and regulators. The compromise overall improved outcomes and reduced harm. While the revised policy might not have been perfect, it was better than a one-sided approach that ignored the interests and priorities of patients. This type of approach would likely have resulted in patients continuing to seek unproven treatments, compromising studies and putting patients at more risk over the long-term.

Unfortunately, the activism around HDC/ABMT for advanced breast cancer did not have similarly positive results and caused more harm than benefit to patients. Favorable court reaction to desperately ill patients, their families, and physicians is understandable. Powerful forces, including the media and lobbying groups, lined up behind patients, often young women, whose stories were compelling [50-52, 58]. The presumption that HDC/ABMT was beneficial shaped much of the public discourse even though it still was unproven [55]. Scientists argued that it was not yet possible to establish the safety and efficacy of HDC/ABMT for advanced breast cancer because more evidence was needed. The courts, on the other hand, held that it was necessary to decide the cases before them using the currently available evidence, which was based on positive results of the therapy in other cancers. Differences in how scientists and courts approve and use evidence to make decisions are well documented [59]. Although the courts were inconsistent, in many cases the plaintiffs won the sympathy of judges and juries, and fear of litigation led others to cover HDC/ABMT [60]. The scientists, clinicians, regulators and advocates failed to work together to compromise on a policy which would have accelerated testing of HDC/ABMT and thereby prevented some of its unnecessary use.

In the end, the HDC/ABMT court rulings harmed patients who received the intervention, led more patients to receive the intervention than would have received it had randomized controlled trials been completed in a timely fashion, delayed answering questions about the safety and efficacy of HDC/ABMT, and cost significant amounts of money [54].

The similarities between HDC/ABMT treatments for breast cancer and SCBIs are striking and could predict the future. Neither intervention requires access to drugs from pharmaceutical companies. Everything is available to clinicians and patients. SC tourism advocates have not yet started using the courts to secure coverage for unproven interventions. Unfortunately, the fact that many patients are obtaining unproven SCBIs abroad rather than participating in the process of generating new knowledge about the safety and efficacy of these interventions through clinical trials poses the same kinds of risks to patients and society as did the pursuit of HDC/ABMT for advanced breast cancer. These patients are circumventing the scientific process of evaluating the safety and efficacy of new interventions. Even if they perceive a positive change in their condition, their treatment outcomes cannot be used to determine safety and efficacy because the procedure is not well-documented and there are no controls or long term evaluations.

SC scientists worry about the impact granting access to unproven treatments may have on the field, especially philanthropic efforts to fund research. In addition, with limited resources including time and funding, scientists are cautious about testing therapies where the preliminary data are limited and not very encouraging. Many worry that if they had ready access to unproven interventions, patients would, like HIV/AIDS and HDC/ABMT patients did, avoid clinical trials with the chance of getting standard care or a placebo. Unfortunately, unproven SCBIs are being marketed and used, particularly through SC tourism. Providing the kind of access may render it very difficult to pursue clinical trials.

### Addressing SC tourism through policy change

The practice of more and more patients with debilitating illnesses going abroad to less developed medical infrastructures to seek SCBIs raises serious problems that should be addressed as a matter of public policy. Since 2008, scientists have worked to educate the public about the risks associated with receiving unproven SCBIs. But this seems to have had little impact on what appears to be a growing market [15]. Other education efforts to change behavior and opinion related to medical interventions, such as vaccinations, also have had little effect [61]. In fact, initial research found that patients were wary of researchers and regulatory systems within their countries—a barrier which cannot be overcome by educational materials from these institutions [26].

Fixing the broken system requires more than education. Educational efforts to combat SC tourism for patients and clinicians should continue and be strengthened, but alone they are unlikely to be sufficient, largely because of the power of hope [9, 26, 27]. The development of adequate policies and regulations to connect, acknowledge and recognize the interests of the stakeholders—the patients, the researchers, the regulators, and the investors—is required. If SC tourism is not addressed as a collaborative effort, policy could be developed through the courts. Unfortunately judges and juries have limited scientific knowledge, different obligations and goals than policy makers, and a propensity to be swayed by persuasive and emotional arguments.

We introduced two examples in which advocates sought access to unproven interventions. These situations highlight how different approaches to patient advocates can lead to better or worse outcomes for all parties. An appropriate and thoughtful policy response that advances multiple competing goals is needed. This insight leads to several questions regarding SC tourism: How is an effective policy response developed? How can we engage patients eager to seek unproven SCBIs outside of US clinical trials and the US health care system, as well as clinicians and scientists eager to study and provide these interventions to develop an effective response to SC tourism? How can policy move from a one-size-fits-all approach to medical regulation to an approach that acknowledges differences among interventions as well as the conditions of patients/research participants? We begin by articulating the principles and goals that should guide policy development and then discuss factors impacting policy development, including the ability to engage stakeholders and create compromise.

### Principles for developing policy

SC tourism can be viewed as a unique issue, but many of the specific ethical and regulatory concerns are similar if not the same as other areas of clinical research, such as the HIV/AIDS and breast cancer cases. When developing public policy, four general principles—that can apply more broadly to biomedical research as well—should be considered: respecting the patient, protecting public health, fostering trust in health institutions, and promoting rigorous clinical research. While these could be applied to developing any health-related policy, we note special considerations related to SCBIs.

First, the policy should respect the interests of seriously ill patients and the authority of individuals to make decisions about what choices are reasonable for them. The interest of the state to restrict access to experimental interventions competes with the interest of patients to pursue possible treatment. This is particularly true for patients with serious or fatal conditions for whom no effective treatments are available.

In developing policy regarding SCBIs, this could mean, for instance, assessing trials and access to interventions for patients with debilitating or fatal diseases differently from others. The interest of future patients should not be favored over those of current patients facing serious or catastrophic disease [62].

Changes to current regulatory policy might include granting more people access to interventions earlier in the trial process, but preventing any access outside of rigorous studies. Alternatively, new regulation could allow studies to move from phase 0 or 1 to phase 2 earlier. Perhaps the FDA could even allowing marketing of therapies earlier, similar to the new policy Japan where SCBI can be marketed during the phase 3 trials [63]. The FDA also might consider initiating clinical trials with less preliminary data, considering different study designs from those typically employed, or utilizing trial designs that avoid ineffective standard treatment arms.

For instance, in 2014 during the Ebola epidemic in Africa, the need to design studies to evaluate both preventative measures and treatments for Ebola generated significant discussion over appropriate trial design. Issues explored included the pros and cons of different designs, such as randomized controlled trials, cluster randomized controlled trials, and studies that use different types of controls, as well as how to identify control groups [64–67]. The scientific merit of different designs is an important consideration, as is the effect different designs have on the willingness of patients and health care professionals to participate and to trust the research process [68].

Second, the policy should protect the health and safety of individuals as well as the public. Achieving this goal involves both facilitating access to promising therapies and preventing access to unsafe ones. Providing patients with interventions, including SCBIs, whose safety and efficacy are unknown puts patients at risk of harm and can lead to a waste of resources. Policy efforts should not treat unproven SCBIs as anything other than experimental interventions. Policy efforts should aim to prevent possible harms and facilitate research that can lead to advances promoting health and efficiency. Therefore, a policy should maximize potential benefit and minimize risks to patients and society. This is an accepted requirement in clinical research [69].

The recent court case *Abigail Alliance for Better Access to Developmental Drugs v. von Eschenbach* and current push for state “Right to Try” laws highlight the tension between the first and second goals. In the Abigail Alliance case the Courts ultimately disagreed, but the Alliance argued that terminally ill patients have a privacy and liberty interest in accessing experimental drugs and that government interference with such access violates patients’ constitutional rights [70]. In response to the loss in the courts, advocates focused their attention on state legislatures by promoting “Right to Try” laws, which allow terminally ill patients access to experimental therapies after they have passed phase 1 trials [71].

Other advocates have offered autonomy-based arguments supporting the right of individuals to be free of FDA interference [27, 72]. But the individual’s right may be tempered by a compelling state interest. The state’s interests identified typically involve protecting people from using harmful products in light of the limited information the consumer might have about a product [73]. Whether such a compelling state interest exists is debated [74]. Policy developed to address SC tourism must reflect an appreciation of this tension.

A third goal is to promote well-placed trust in physicians and health care institutions. Trust in the physician-patient relationship is important because it is necessary for an effective therapeutic alliance [75]. As such, “preserving, justifying, and enhancing trust is the fundamental goal of much of medical ethics, and is a prominent objective in health care law and public policy” [75]. The need for consumers to be able to rely on the information they receive about medications shapes FDA requirements for truth in advertising. These restrictions limit what companies may assert about their products [76]. This helps to promote trust and gives patients assurance that the claims made by companies are supported by evidence. But the FDA could do more to police these websites by engaging the public in their efforts with easy and anonymous online reporting of fraudulent clinics and websites.

In addition to the company websites and advertisements, physicians need to be aware of the problem and risks associated with recommending unproven SCBIs. This is especially true of the celebrity doctors, who make health recommendation with little to no scientific data to back them up [77]. Policing doctors’ practice is traditionally the role of state licensing boards, but policy makers have started to review the problem even calling a celebrity doctor in front of Congress to defend his on camera comments related to health products [78].

Finally, policy should promote ethical and rigorous scientific research. The importance of the ethical conduct of research is widely recognized [79–81]. Policy should also promote scientific progress or at least not impede it unless necessary for public safety. It should protect research from inappropriate influences and biases. Moreover, “science policy should support the needs of citizens” [62, 82]. Whatever policy is developed, it has to ensure data validity and integrity. Without reliable data, people remain at risk, science does not advance, money and other resources are wasted, and effective treatments will remain elusive. Requiring a system for thorough documentation throughout the SCBI research process, including follow-up post-treatment evaluation will facilitate long-term progress in identifying risks and increasing efficacy. If changes are made to make allowances for earlier access to SCBIs, this must be done in connection with continued rigorous research to determine the efficacy and safety of the intervention.

Advancing these four goals will help create a stronger, more effective policy that reflects stakeholder needs and priorities. Policy proposals should acknowledge these goals and be tested against them. This is challenging both because of the tensions among these principles and because policies adopted in the US only shape US practices. SC tourism is evidence of the problems that emerge when different practices and policies prevail in different areas. While international organizations, such as ISSCR, continue to discuss and pass guidelines which prohibit the use of SCBIs outside of a clinical trial, they are non-binding and cannot force clinics to close.

Compromises will most likely be needed to obtain the necessary data to refute or validate claims of SCBI clinics and hopefully in the long run help promote public and individual health. But making these compromising could impact the quality of research in the short term.

### Factors to develop solutions

A SC tourism policy solution should integrate the desire to innovate and to provide and receive experimental interventions with the need to pursue research and evaluate safety and efficacy. The FDA response to SC tourism needs to involve engaging stakeholders, especially patient advocates, scientists, regulators, and clinicians, in robust consultation processes. In those processes, the priorities and concerns of each group should be identified and various types of changes should be considered. From what we know now, the underlying reasons for SC tourism include frustration, desperation, varying accounts of truth, and hope [9, 27]. Patient advocates, scientists, regulators, and clinicians have competing interests and goals [64]. Part of the stakeholder engagement process should focus on overcoming these obstacles and identifying common goals and ways to achieve them.

The idea of engaging stakeholders to advance science and solve problems is not new. The National Institutes of Health (NIH) has established community engagement and community engaged research as priorities in multiple ways [83]. Much can be learned from these efforts to include stakeholders in identifying research questions, designing and conducting studies, and interpreting and disseminating data. Methods developed to engage stakeholders by NIH and NIH-funded scientists and others can be adopted to develop a policy response to SC tourism. Methods used in Patient-Centered Outcomes Research Institute (PCORI) studies might be especially useful [84]. These include the use of stakeholder panels to identify research priorities and aims; to contribute to and evaluate study design; to develop communication materials such as consent forms; to assess research risks and burdens and propose ways to mitigate these; to evaluate the information that should be communicated to participants during and after a trial, to create decision aids, and to address problems or questions that emerge during a study.

Other examples of ways to engage stakeholders include stakeholder-specific in-person and virtual town hall meetings and focus groups. Such gatherings may be aimed at identifying priorities and concerns, generating new policy or other ideas, and evaluating existing ideas for policy change [84]. The Robert Wood Johnson Foundation also has developed community engagement resources that could be adapted to address SC tourism [85]. These include community boards that participate actively in identifying problems, crafting policies and interventions to address those problems, and implementing and evaluating changes. It might also be appropriate to ask groups to generate white papers or other documents that can help advance the conversation. Bringing stakeholder groups together for dialogue may facilitate creative and effective solutions. One model for achieving this is the Bob Woodruff Foundation’s High Impact Collaboration Series, which is designed to identify specific problems and solutions by bringing together key stakeholders and contributors who can promote effective changes [86]. Another way to establish consensus within and across stakeholder groups is the use of Delphi panels [87] Delphi panels could be used to rank research priorities, or identify the most acceptable compromises or study designs.

Knowledge and new insights gained from stakeholder engagement should inform policy development. Another group of stakeholders who should be consulted included representatives from clinics offering unproven SCBIs. While some clinics may not be trustworthy and some may have no interest in participating in the clinical trial process, others could be encouraged to initiate a form of a trial to collect data in a manner that is still overseen or regulated by the FDA. The FDA should work to integrate (some) clinics offering unproven interventions and procedures so data can be collected into a form of clinical trials [23, 88]. But this requires changes in FDA policies. Many innovative SCBIs lack data—positive or negative. Currently, the FDA requires that investigators and companies demonstrate that they have sufficient information “to assure the safety and rights of subjects” (21CFR312B.22(a)) to justify human testing. So, while they are subject to FDA oversight and their products require FDA approval, many SCBI clinics cannot obtain permission from the FDA to initiate trials. This has led patients and doctors in some cases to move clinics outside of US so they can continue the interventions. If the FDA maintains its current position, people will continue to obtain unproven SCBIs outside of US and the effectiveness of interventions will continue to be unknown.

We should be wary about making any radical policy changes to the FDA clinical trial and approval process. Expanding FDA clinical trials needs to be done carefully. Access to untested interventions could result in harm including unfinished clinical trials (because patients are unwilling to volunteer), less rigorous trials and overall harm for society. Furthermore, some critics note that the FDA is not well suited to oversee SCBIs because cells differ in multiple ways from drugs and biologics FDA policies were designed to regulate. SCBIs use cells which are not cleared from the body like drugs are and the interventions used are often patient-specific. But the FDA has a long history of adapting to new technologies and already has been developing pathways for regulating personalized medicine interventions including drugs for specific disease-causing mutations (such as Kalydeco) and autologous vaccines (such as Provenge) [89].

Any regulatory approaches developed to address SC tourism must be clear and transparent [90]. And they must be designed in a way that recognizes that not all innovative interventions turn out to be safe and effective treatments. It is problematic to assume that everything new is good or at least will not be harmful, or that something always is better than nothing. New regulatory approaches also should be evaluated to determine whether they are effective. Metrics to evaluate new and existing policies should be identified and employed in policy assessment. This can include evaluating completion of clinical trials, effectiveness of SCBIs, the extent to which SC tourism decreases, and necessity of the policy if it’s determined that the SCBIs are unsafe or ineffective.

## Conclusion

In 2004, patient advocate groups were major players in helping implement and pass significant public policy and funding initiatives in SCs and regenerative medicine. In the following years, advocates encouraged policy makers to broaden embryonic SC research funding, which was ultimately passed after President Barack Obama came into office in 2009. Many advocates did this because they were told SC research would lead to cures. After waiting more than 10 years, many of these same patients are going to clinics around the world offering experimental SCBIs against the advice of the majority of doctors and SC scientists.

A policy intervention to reduce SC tourism is needed. It must address the needs and concerns of the major stakeholders: patient advocates, researchers, clinicians, and regulators. It should be an evidence-based policy, i.e., one grounded in an understanding of the competing interests and priorities at stake and in an analysis of the effects of different approaches. Metrics for evaluating the success of these policies should be established in advance and data gathered over time. Policies should be changed if found ineffective.

It remains to be seen what would be the best and be most appropriate model for addressing SC tourism. Nevertheless, change is needed. Previous ways advocates pushed for change, with both positive and negative results for the patients, can help guide how SC tourism is approached by US policy makers. Working as a group to formulate a compromise policy addressing the needs of different stakeholders will be the only way to curb the growing and disturbing trend of SC tourism.

## Abbreviations

ABMT: autologous bone marrow transplant
CIRM: California Institute of Regenerative Medicine
FDA: US Food and Drug Administration
HDC: high-dose chemotherapy
IND: investigational new drug
ISSCR: International Society for Stem Cell Research
JDRF: Juvenile Diabetes Research Foundation
NIH: National Institutes of Health
PCORI: Patient-Centered Outcomes Research Institute
SC: stem cell
SCBI: stem cell-based intervention
